# Experimental models for analysis of oligodendrocyte pathophysiology in stroke

**DOI:** 10.1186/2040-7378-1-6

**Published:** 2009-10-24

**Authors:** Ken Arai, Eng H Lo

**Affiliations:** 1 Neuroprotection Research Laboratory, Massachusetts General Hospital, Harvard Medical School, Charlestown, USA

## Abstract

White matter damage is a clinically important part of stroke. However, compared to the mechanisms of neuronal injury in gray matter, white matter pathophysiology remains relatively understudied and poorly understood. This mini-review aims at summarizing current knowledge on experimental systems for analyzing the role of white matter injury relevant to stroke. In vitro platforms comprise primary cultures of both mature oligodendrocytes (OLGs) as well as oligodendrocyte precursor cells (OPCs). Tissue platforms involve preparations of optic nerve systems. Whole-animal platforms comprise in vivo models of cerebral ischemia that attempt to target white matter brain areas. While there is no single perfect model system, the collection of these experimental approaches have recently allowed a better understanding of the molecular and cellular pathways underlying OLG/OPC damage and demyelination. A systematic utilization of these cell, tissue and whole-animal platforms may eventually lead us to discover new targets for treating white matter injury in stroke and other CNS disorders.

## Introduction

Over the past decade, impressive advances have been made in understanding the basic molecular mechanisms underlying neuronal death. However, clinically effective neuroprotectants have not yet been discovered. This has been especially true in stroke, where many drugs targeting excitotoxicity, oxidative stress, and inflammation have all failed [1-3]. Although there are many difficult reasons for these translational problems [4-6], one potential issue worth examining further is the lack of emphasis on white matter.

In the CNS, white matter is primarily comprised of axonal bundles ensheathed with myelin. The cells forming these sheaths are the oligodendrocytes (OLGs), which tend to be arranged in rows parallel to axonal tracts. Just before and after birth, oligodendrocyte precursor cells (OPCs) multiply rapidly, mature into OLGs, and develop processes, which are then involved in the formation of myelin. Damage to OPCs and OLGs causes loss of myelin synthesis and interruption of proper axonal function. Hence, even if we protect neurons in gray matter, loss of myelin and axonal integrity would interfere with neuronal connectivity and function. Neuroprotection cannot be truly attained without oligoprotection.

White matter ischemia is different from gray matter in many ways. For instance, white matter ischemia is typically more severe than gray matter because white matter blood flow is lower than gray matter and there is little collateral blood supply in deep white matter [6]. Moreover, mechanisms responsible for ischemic cell death may be different between white matter and gray matter. Although an increase in intracellular Ca2+ is involved in both white matter and gray matter ischemia, the routes of Ca2+ entry might differ. In white matter, pathological Ca2+ entry occurs in part due to increased intracellular Na+ and membrane depolarization [7-9]. Therefore, Na+ channel blockade has been proposed as a protective strategy in white matter [7-10]. In contrast, voltage dependent Ca2+ channels and glutamate-activated ionic receptors in gray matter have been traditionally viewed as the primary routes of pathological Ca2+ entry [11,12]. Of course, beyond these differences in calcium handling, there are potentially many other aspects of gray versus white matter function that differ. Thus far, white matter pathophysiology remains relatively poorly understood compared to gray matter. Because white matter damage is a clinically important part of stroke, we might need to take white matter ischemia more into account to develop effective stroke therapy.

In this mini-review, we summarize the current knowledge on experimental systems for assessing white matter damage by ischemic stress. A systematic utilization of these cell, tissue and whole-animal platforms should enable us to better dissect mechanisms of white matter pathophysiology and help our search for oligoprotectants in stroke and other CNS disorders.

## In vitro white matter ischemia models

In pathological conditions, excitotoxic cell death is a critical part of neuronal injury, and it has been implicated in acute injury to the CNS and in chronic neurodegenerative disorders [13-15]. Numerous studies have now revealed that, in addition to neurons, glial cells can also be damaged by excitotoxicity. Among the glial cell types, the OLG lineage may be the most vulnerable to excitotoxicity.

In the gray matter of the brain, neuronal cell death is often caused by a rise of extracellular glutamate concentration, which activates NMDA receptors and leads an excessive rise of intracellular Ca^2+ ^concentrations. Glutamate can also damage white matter OLGs, in both acute and chronic diseases [16]. Since excessive glutamate release can be seen under brain ischemic conditions, glutamate-induced OLG death has been used as a model for in vitro white matter ischemia to pursue the mechanism of OLG death by excitotoxicity. So far, there are at least three different mechanisms of glutamate-induced OLG death. Using cultured OLGs prepared from new born rat brain, Oka et al. reported that OLGs in cultures are highly vulnerable to glutamate. They showed that 24-h exposure of glutamate caused OLG death by reversing cystine-glutamate exchange, which induces glutathione depletion [17]. AMPA/kainate receptors have also indicated to be involved in glutamate-induced OLG death. Sanchez-Gomez et al. used primary cultures of OLGs derived from the optic nerves of young-adult rat or mouse to examine the involvement AMPA/kainite receptors in OLG death. In the report, they revealed that excessive activation of AMPA/kainate receptors causes Na^+ ^and Ca^2+ ^influx through the receptor channel complex leading to OLG death [18]. Until recently, it had been thought that NMDA receptor was not involved in OLG death by excitotoxicity. However, three recent studies have demonstrated that OLGs also express NMDA receptors as neurons [19-21]. NMDA receptors of OLGs are activated by glutamate in white matter ischemia [19], and activation of these receptors leads to rise of intracellular Ca^2+ ^concentration [21]. Hence, NMDA receptors might be also participate in glutamate-induced OLG damage.

In addition to these primary excitotoxic mechanisms, glutamate can also kill OLGs via immune-system-related pathways. Alberdi et al. showed that brief incubation with glutamate followed by exposure to complement is lethal to OLGs in vitro [22]. Thus, even glutamate at non-toxic concentrations can kill OLGs by sensitizing these cells to complement attack. Further, inflammatory cytokines such as TNF-alpha and IL-1beta are also known to be involved in glutamate-induced OLG death. Those cytokines are released by reactive microglia and can impair glutamate uptake and trigger excitotoxic OLG death [23]. Since glutamate is reported to release TNF-alpha from microglia through AMPA/kainate activation, glutamate can also induce OLG death in indirect mechanisms [24].

Although elevations in glutamate certainly occur in stroke, alterations in other extracellular mediators may also be important. In the CNS, extracellular ATP can act as an excitatory neurotransmitter. ATP activates ionotropic P2X receptor and metabotropic P2Y receptor [25,26]. Both P2X and P2Y receptors are expressed in OLGs. James and Butt used isolated optic nerves to show that ATP increased intracellular Ca^2+ ^concentrations in OLGs through P2Y receptors [27]. In addition, they also demonstrated that a P2X receptor agonist evoked a smaller but significant OLG Ca^2+ ^signal. In brain ischemia and spinal cord injury models, P2X receptors were reported to mediate signaling cascades leading to neurodegeneration [28,29]. Also in OLGs, P2X was shown to be involved in cell death. Matute et al. have demonstrated that ATP or P2X agonists, but not P2Y agonists, was toxic to differentiated OLGs in vitro [16].

No matter what the upstream trigger might be, energetic stress should be a common denominator for white matter injury. In this regard, oxygen-glucose deprivation (OGD) is a useful tool for mimicking in vitro ischemia. OGD can induce both OPC as well as OLG death in vitro [30,31]. Indeed, it appeared that OPCs were more susceptible than OLGs [30]. More recently, using an immortalized mouse OLG cell line, Zhang et al showed that OGD-induced OLG death was induced by apoptosis via p75 and caspase-3 [32]. Thus, these cell systems can be productively used to dissect the molecular pathways of OGD-induced white matter injury.

Compared to astrocytes, both OLGs and OPCs are relatively weak. To maintain those cells in culture, a wide spectrum of culture supplements such as growth factors are needed. Hence, removing those factors from culture media causes OLG or OPC death. Although this starvation stress does not perfectly reflect in vivo ischemic conditions, the stress can be thought as one of the in vitro ischemia model and is now well used to induce OLG/OPC damage, especially for OPC cultures. For instance, Cui et al. reported two studies whereby IGF-1 promoted the proliferation of OPCs via PI3K/Akt, MEK/ERK, and src-like tyrosine kinase pathways [33,34]. Rubio et al. showed that neurotrophin-3 is also important factor for OPC survival [35]. GGF/neuregulin has been also reported to be OPC protective [36]. Taken together, these serum deprivation paradigms provide evidence for the essential nature of trophic coupling for oligoprotection. Indeed, these types of growth factor gain-and-loss experiments may help us test drugs that can be used to salvage OPC and OLG health in a wide range of disorders.

The experimental systems described above are all useful for examining the mechanisms of OLG/OPC death by ischemic stress. The main read-outs comprise either cell survival or myelin synthesis in isolated cultures, or compound action potentials in optic nerve preparations. The ease of quantitation and the reproducibility of culture platforms might allow for relatively high-throughput screening. However, it must be acknowledged that in vitro systems have some limitations. Cell and tissue systems cannot truly replicate the inter-cellular interactions and anatomic geometry of in vivo white matter.

## In vivo rodent white matter ischemia models

White matter lesions are observed frequently in stroke patients and experimental animal models of cerebral ischemia, and have been thought to contribute to cognitive impairment [37-39]. Since nonhuman primates have well-developed white matter and vascular architectures which are similar to those of human brains, it seems to be reasonable to use them for studying the mechanisms of white matter injury. However, from ethical and practical standpoints, there remains an important imperative for developing rodent models. Compared to in vitro studies of white matter injury, basic research of white matter injury via in vivo models is not as well developed.

Some research groups have shown that OLG damage occur in response to ischemia in rodent middle cerebral artery occlusion (MCAO) models. Irving et al. observed structural changes of the OLG cytoskeleton by 40 min occlusion of MCA [40]. They assessed these changes by detecting increase of tau immunoreactivity within OLGs. Tau is the microtubule associated protein, and the increase in immunoreactivity has been found to be a sensitive marker for OLG damage. The MCAO-induced OLG damage assessed by tau immunoreactivity has been confirmed by other groups [41,42]. Axons and myelin structure have also shown to be damaged in rodent MCAO models [43]. Another report by Irving et al. carefully examined the methods of quantifying white matter injury following prolonged focal cerebral ischemia in the rat. This study showed that myelin basic protein, Tau 1, and amyloid precursor protein staining can be utilized to assess myelin and axonal integrity in rat MCAO model [44]. Schabitz et al. have also shown the white matter injury in rat stroke model [45]. The use of Luxol-fast blue-periodic acid-Schiff and Bielschowsky's silver impregnation allowed the detection of myelin and axons, which could then be semi-quantified as read-outs for specific experiments.

The two-vessel occlusion model by permanent bilateral occlusion of the common carotid arteries has also been used to induce white matter ischemia. This model produces chronic cerebral hypoperfusion in rat and gerbil [46,47]. These models are characterized by pathological changes in white matter, which appear similar to those in human cerebrovascular white matter lesions [48,49]. As seen in human white matter ischemia, the rat chronic cerebral hypoperfusion model by the ligation of the bilateral common carotid arteries is accompanied by cognitive impairment [50]. In this model, however, the visual pathway is also injured by the occlusion of the ophthalmic arteries, and thus may affect behavioral assessment [47]. Although rats and gerbils are mostly used for these chronic cerebral hypoperfusion models, mice have also been used recently. With newly designed micro-coils, Shibata et al. developed a new mouse model of chronic cerebral hypoperfusion with relative preservation of the visual pathway [51]. In this model, white matter lesions occurred after 14 days without any gray mater involvement. Another report from Shibata et al. has demonstrated that the mouse hypoperfusion model showed impairment of working memory using the 8-arm radial maze [52]. The importance of the mouse model centers on the ability of future studies to utilize specific knockouts or transgenics for dissecting molecular mechanisms and mediators. For example, the use of knockout mice for matrix metalloproteinases (MMPs) could be anticipated. From a biochemical basis, MMPs can directly attack myelin components [53]. In fact, Nakaji et al. used the mouse chronic cerebral hypoperfusion model to show that gene knockout of MMP-2 reduced the severity of the white matter lesions [54]. Furthermore, MMP-9 knockout mice showed the resistance to MCAO-induced degradation of myelin-basic protein [55]. Depending on the proposed mechanisms, the combination of specific knockout mice with experimental induction of stroke or brain injury should be useful for exploring the underlying mechanisms involved in white matter pathophysiology.

In addition to the methods of occluding blood vessels, the direct injection of endothelin-1 (ET-1) into the neural parenchyma has also been used to induce white matter ischemia [56]. ET-1 is a potent vasoconstrictor peptide, and acts through different receptors called types A and B, which are distributed throughout the CNS [57-59]. Hughes et al. reported that microinjection of ET-1 into the striatum and cerebral cortex induces focal ischemia with a reduction of 40% of local blood flow in rats [60]. The damage in this model is localized in both gray and white matter without blood brain barrier breakdown. Frost et al. also used ET-1 microinjection model to induce white matter ischemia [61]. They injected ET-1 into the internal capsule, and tissue necrosis and demyelination in the infarcted white matter were found after a 14-day survival period. Further, infarcts resulted in measurable sensorimotor deficits. Regarding inflammatory response in the ET-1-injection white matter ischemia model, two studies recently came out. They showed that inflammatory response and white matter damage are closely related in the rat models of ET-1 microinjection into the striatum [62,63].

The optic nerve has been thought as one of the most useful regions to study OLG functions. As cited in the previous section, numerous groups use OLG cultures from rodent optic nerve to investigate the OLG death by ischemia. Also in vivo system, the mechanisms of OLG damage have been examined in optic nerve. Retinal ganglion cell (RGC) axons comprise the optic nerve. So far, several in vivo models have been created to identify the RGC response to optic nerve damage [64-67]. A recent effort used photochemically-induced thrombosis to evoke anterior ischemic optic neuropathy (AION) in rats and mice [68,69]. AION may mimic optic nerve strokes, which are among the most common causes of sudden optic nerve-related vision loss. Discrete histopathological changes of OLGs were reported in human AION [70]. Correspondingly, OLG dysfunction was observed in the mouse AION model [69].

Taken together, the emergence of various mechanical, thrombotic and chemical models now provides reasonable ways to move forward in vivo. But it must be acknowledged that white matter volumes in rodents are extremely small compared to human brains. Thus, methods for quantifying outcomes are challenging. Ultimately, comparisons of these rodent outcomes with human stroke patients must be performed carefully since white matter connectivity may be different between these vastly differing levels of brain evolution.

## Remyelination in recovery phase in rodent stroke models

To date, the majority of studies using cell, tissue and whole-animal models of white matter injury have mostly focused on mechanisms and targets for acute injury. However, it may not be easy to block all multifactorial pathways of brain cell death in stroke patients. Therefore, an emerging emphasis on promoting recovery after stroke is beginning to take shape in our field. In the context of white matter mechanisms, it will be important to carefully define how each of the model platforms can be applied in this regard.

To support normal brain function, neuronal connectivity must be maintained. Hence, remyelination and regenerating axons in the border zone of cerebral infarcts and in secondarily lesioned areas are essential for stroke recovery. Fundamental research into these mechanisms of remyelination after ischemia remains somewhat limited, but a few studies are beginning to lead the way. Mandai et al. reported that myelin repair can occur in peri-infarct areas in mouse MCAO model, as judged by upregulated gene expression of proteolipid protein, one of the major protein components of CNS myelin [71]. Gregersen et al. studied the expression of myelin basic protein (MBP), another major component of CNS myelin, in peri-infarct areas using rat MCAO model [72]. They revealed that peri-infarct OLGs increased their expression of MBP mRNA from 24 h to maximal levels at day 7. These changes corresponded to the appearance of process-bearing MBP and occasional myelin oligodendrocyte glycoprotein-immunoreactive OLGs in parallel sections.

As described before, OLGs originate from their precursors; OPCs. OPCs are now found not only during development but pockets might also exist throughout the adult brain [73-76]. Therefore, OPCs may contribute to myelin maintenance and repair by generating new OLGs as a source of remyelination and repair. Experimental evidence is starting to be collected showing that OPCs in adult brain contribute to replenishment of OLGs after ischemic insults. Tanaka et al. examined the alteration of OLGs, OPCs and myelination in rat MCAO model [77]. They showed that a rapid and progressive decrease in the number of OLGs, OPCs, and myelin density after 2-day in the infarct core. In contrast, the peri-infarct area exhibited a moderate reduction in the number of OLGs and the myelin density with a slight increase in OPCs at 2 days after MCAO. Subsequently, a steady increase in the number of OPCs and a gradual recovery of OLGs were found in the peri-infarct area at 2 weeks of MCAO.

If OPCs provide a source of potential white matter repair, what signals and substrates are involved? A recent interesting study by Komitova et al. suggested that enriched environments may enhance the generation of OPCs after focal cortical ischemia. In this study, newly born OPCs were found to be immunoreactive for brain-derived neurotrophic factor (BDNF) [78]. Thus, it is tempting to speculate that BDNF may be related to remyelination by OPC proliferation/differentiation. What remains to be clarified is how this signal may interact with a multitude of other growth factors involved in OPC proliferation/differentiation. Since growth factor expression is upregulated after ischemia in peri-infarct area, a network response of growth factors might have essential roles in remyelination after stroke. These studies have mostly utilized in vivo models. As we dig down deeper mechanistically, a systems biology approach may ultimately be required as one asks broadly what gene profiles mediate cell-cell interactions in white matter during and after injury.

## Potential therapeutic targets for white matter ischemia

**Figure 1:**
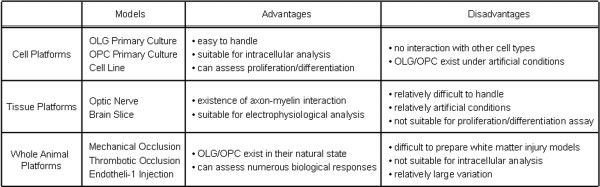
**Schematic for experimental systems and their endopoints**. All systems have both advantages and disadvantages. A systematic utilization of these systems should enable us to better dissect mechanisms of white matter pathophysiology and help our search for oligoprotectants in stroke and other CNS disorders.

**Figure 2:**
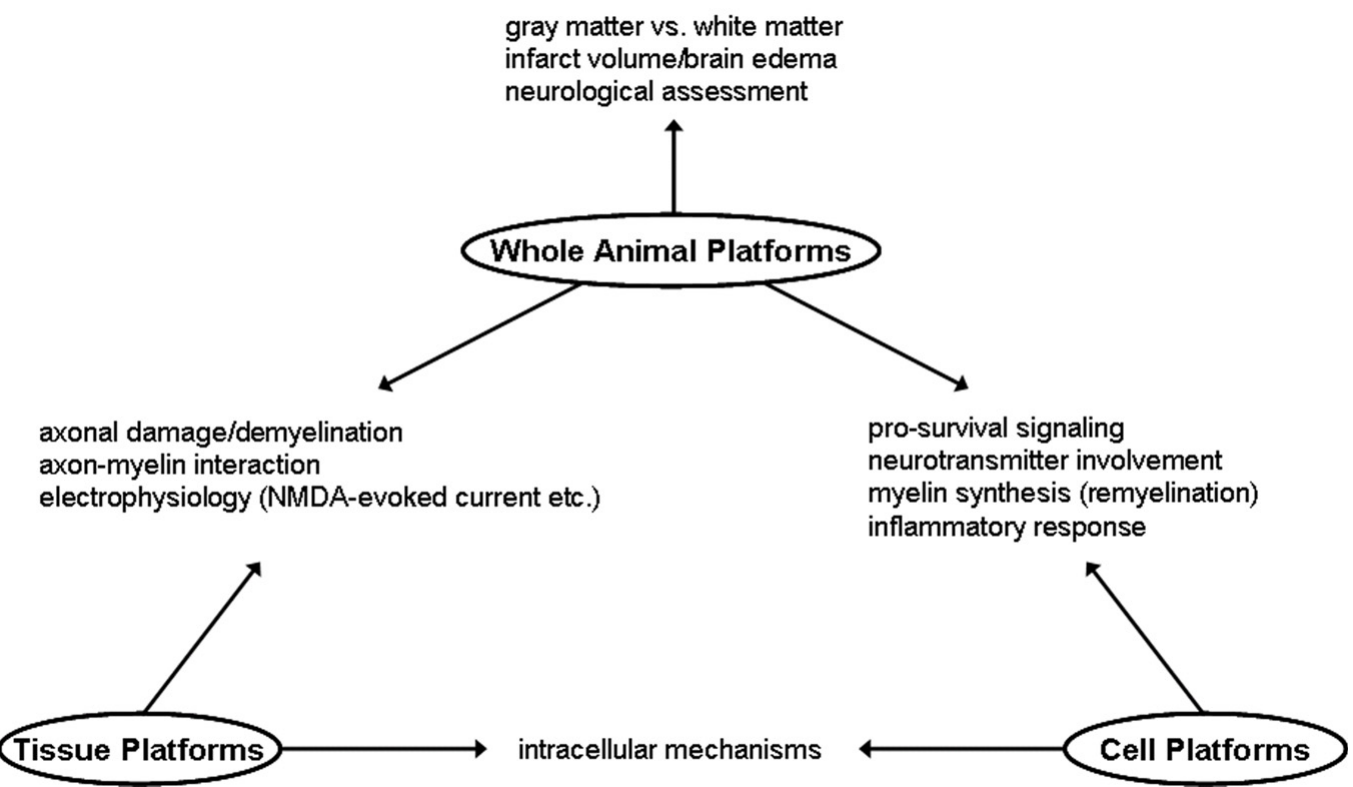
**Summary for experimental systems for analyzing the role of white matter injury relevant to stroke**.

The combined use of cell, tissue and whole-animal platforms discussed here may provide powerful tools for dissecting the pathophysiologic mechanisms of white matter injury in CNS disorders (see Figs. 1 and 2). Of course, no validated drugs yet exit. But several promising leads should be discussed.

Memantine, an uncompetitive NMDA receptor antagonist, has been well examined for the efficacy for preventing white matter from several insults including ischemic stress. Memantine is now licensed for moderate-to-severe Alzheimer's disease in US and EU [79]. Very recently, two studies suggested that memantine may be protective to white matter. Bakiri et al. reported that memantine reduced ischemic damage to mature and precursor OLGs in brain slices assessed by patch-clamp system [80]. Manning et al. showed that memantine attenuated white matter injury in a rat model of periventricular leukomalacia [81]. Therefore, NMDA receptor antagonists might be a good target for white matter injury after stroke.

Although the radical spin-trap NXY-059 failed in clinical stroke trial recently [82], antioxidant drugs are still potent therapeutic candidates for white matter. Imai et al. used rat transient ischemia models to evaluate the efficacy of ebselen, an anti-oxidant drug [41]. In this study, they showed that ebselen reduced axonal damage, and that OLG pathology was also reduced. Using rat MCAO model, Irving et al. have demonstrated that a free radical scavenger phenyl-N-tert-butyl-nitrone (PBN) reduced the number of tau-positive OLGs in the subcortical white matter of the ischemic hemisphere [40]. Lin et al. also examined the efficacy of PBN on white matter injury by hypoxia-ischemia in the neonatal rat brain [83]. In the study, the PBN treatment protected both OLGs and axons from ischemic insults. Subsequently, the same group has demonstrated that PBN also inhibited up-regulation of inflammatory cytokines such as IL-1beta, TNF-alpha and iNOS mRNA expression in the same model [84].

Finally, an ongoing NIH-funded trial is assessing minocycline for acute stroke. Minocycline is a second-generation tetracycline, which can cross the blood-brain barrier [85,86]. Minocycline has been shown to be beneficial in a wide range of acute neurological injuries. In rodent brain ischemic models, this drug showed anti-inflammatory effects, based on its ability to inhibit immune mediators such as microglia [87,88]. Although there is no report that minocycline directly protects OLGs against ischemic stress in adult rodent stroke model, this drug was shown to attenuate hypoxia/ischemia-induced white matter injury in the neonatal rat [89,90]. Further, Hewlett and Corbett have shown that delayed minocycline treatment reduced long-term functional deficits as well as white matter injury in ET-1-induced rat ischemia model [91]. In spite of these experimental findings, it must be noted that a recent clinical trail using minocycline in ALS patients failed to show efficacy [92]. A potential caveat with this study is the long-term use of minocycline. Among its many actions, minocycline is a powerful metalloproteinase inhibitor. It has been recently suggested that long-term suppression of metalloproteinases may be detrimental for neurovascular homeostasis [93]. In stroke, short term applications of minocycline may still be possible. Ultimately, however, whether minocycline will be useful for white matter injury in stroke patients will have to be answered in a carefully analyzed randomized trial.

## Conclusion

OLGs, myelin-forming glial cells in the CNS, are very vulnerable to ischemic stress, resulting in early loss of myelin and white matter dysfunction. Inhibiting axonal damage and/or OLG death, and accelerating the remyelination via OPC proliferation and differentiation may turn out to be critical for preventing acute neuronal disconnections as well as promoting repair and remodeling after stroke. Although there are no clinically validated treatments to date, several promising leads are beginning to be dissected in experimental systems. In this mini-review, we tried to provide a broad but brief survey of existing models at the cell, tissue and whole-animal levels. Many studies have productively used these model systems to dissect pathophysiology as well as assess treatment strategies. As we seek to cross the difficult translational hurdles between basic science and clinical challenges, the combined use of multiple model systems should help.

## Competing interests

The authors declare that they have no competing interests.

## Authors' contributions

Study concept and design: KA and EHL; Drafting of the manuscript: KA and EHL; Critical revision of the manuscript for important intellectual content: KA and EHL; Obtained funding: KA and EHL. All authors read and approved the final manuscript.
